# For Better and For Worse: Frequent Gamblers Use Dual Counterfactuals to Justify Continued Gambling

**DOI:** 10.1007/s10899-023-10221-2

**Published:** 2023-06-05

**Authors:** Christina I. Anthony, Elizabeth Cowley, Alex Blaszczynski

**Affiliations:** 1 University of Sydney Business School, Rm 4006, H70, Sydney, NSW 2006 Australia; 2 Faculty of Science, University of Sydney, Sydney, NSW 2006 Australia

**Keywords:** Counterfactual thinking, Frequent gamblers, Affect, Motivated reasoning, Responsible gambling

## Abstract

How might frequent gamblers convince themselves to keep playing despite persistent losses or after a win that should be savored? The purpose of this research is to examine the unexplored question of how frequent gamblers’ use counterfactual thinking to motivate their desire to continue gambling. Using a sample of n = 69 high and n = 69 low frequency gamblers in a field setting, we found that infrequent gamblers tended to consider how the perceived outcome of losing “could have been better” (i.e., upward counterfactual thinking), and how a winning outcome “could have been worse” (i.e., downward counterfactual thinking). This pattern of counterfactual thinking is considered typical in many settings and may, in a gambling context, support a potentially more responsible approach by helping infrequent gamblers to learn from past mistakes to avoid significant future losses and to savor wins to protect returns gained. Alternatively, we found that frequent gamblers were more likely to generate ‘dual counterfactuals’ which include both upward and downward counterfactuals in response to losses and wins. We argue that this dual pattern of counterfactual thinking may allow frequent gamblers to more easily justify their desire to continue gambling. Findings suggest that challenging gamblers counterfactual thinking patterns could assist clinicians in moderating the potential for high-risk behaviors.

## Introduction

A common observation in the gambling literature is that some gamblers embrace risky behavior and continue to gamble despite persistent losses (Blaszczynski & Nower, 2002). In addition to a range of erroneous cognitions and misunderstanding probabilities, ‘chasing’ to recoup losses and the motivation to win more money is commonly found among gamblers (McBride et al., 2010; O'Connor & Dickerson, 2003; Sacco et al., 2011) with research suggesting more than 75% of problem gamblers engage in these forms of behaviours (Toce-Gerstein et al., 2003). This is particularly relevant as chasing is critical in the development and maintenance of gambling disorders (Breen & Zuckerman, 1999; Corless & Dickerson, 1989; Gouiaan et al., 2014; Lesieur & Custer, 1984; Sharpe, 2002). One possibility explaining this persistence may be that individuals experiencing problem gambling endorse an array of irrational beliefs and evaluate outcomes in a biased manner which promotes risky decision-making and excessive gambling (Armstrong et al., 2020). The tendency for gamblers to evaluate their outcomes in a biased manner is consistent with research on motivated reasoning (Kunda, 1990; Lord et al., 1979; Molden & Higgins, 2005) which highlights that people like to believe they are good decision makers and are highly motivated to bias their perceptions to support their beliefs (confirmation bias). In fact, gamblers’ tendency to form biased evaluations frequently occurs even when outcomes are determined by pure chance (Gilovich, 1983).

In this study, we investigated the little understood role of counterfactual thinking and dual counterfactuals in gambling behavior to examine how such thinking can be used in a potentially biased and risky way to justify decisions to continue gambling in the face of emerging gambling problems. In the following section we define counterfactuals and their functionality and then apply it to the case of gambling and problem gambling.

## Functionality of Counterfactual Thinking

Counterfactual thinking is a specific type of conditional reasoning that occurs when people evaluate an outcome by comparing results with other simulated or imagined outcomes which could have occurred, but did not (Roese, 1997). Thoughts such as “what if”, “if only” and “at least”, typically reflect this mode of thinking. Counterfactuals are often categorized by their direction of comparison (McMullen et al., 1995; Roese, 1994). Upward counterfactual thoughts (UCTs) involve the generation of better alternative outcome(s) to reality (i.e., “if only I had studied more, I could have earned an A on the exam”). Downward counterfactuals thoughts (DCTs) involve the generation of worse alternative outcome(s) to reality (“at least I studied a bit, otherwise I might have failed the exam”).

An important determinant of the directionality of counterfactual thoughts is the valence of the outcome. A number of studies have found that people are more likely to spontaneously generate UCTs following failure, and DCTs following success (Gleicher et al., 1995; Grieve et al., 1999; Markman et al., 1993; Sanna et al., 2001). One explanation for this tendency is provided by functional accounts of counterfactual thinking (Epstude & Roese, 2008; Markman et al., 1993; Roese, 1994; Roese & Epstude, 2017; ). Functional accounts (Epstude & Roese, 2008; Roese, 1994; Roese & Epstude, 2017) assume that counterfactuals can beneficially serve people’s goal states and motivations. In particular, researchers agree on two conceptually distinct functions of counterfactual thinking; (1) preparation for the future and (2) affect regulation. Previous research suggests that the preparative function (sometimes referred to as “self-improvement”) is best served by UCTs because they can mobilize increased effort and persistence (Kray et al., 2009; Markman et al., 1993, 2008; Markman & McMullen, 2003; McCrea, 2008; Roese & Olson, 1997) by revealing potential paths to more successful goal outcomes. Specifically, the construction of UCTs enable individuals to identify possible causal inferences for poor performance and goal blockage (Epstude & Roese, 2008). These causal conclusions can lead to corrective strategies to achieve intended outcomes (Roese, 1994; Smallman, 2013; Smallman & Roese, 2009) which should be particularly useful following failure.

Conversely, in the functional approach, DCTs are believed to serve beneficial affect regulation functions (McMullen, 1997; McMullen & Markman, 2000; Roese & Olson, 1997; Roese & Hur, 1997; Taylor & Schneider, 1989; White & Lehman, 2005). This allows individuals to maintain positive feelings or feel better about themselves (referred to as “self-enhancement”), by virtue of the positive affect (or reduction in negative affect) elicited from the affective contrast with the worse alternative avoided (Markman et al., 1993; Medvec et al., 1995; Roese, 1994; Sanna et al., 1999). Therefore, DCTs can enhance enjoyment for a positive outcome by allowing individuals to appreciate their success at avoiding alternative outcomes which could have easily been worse (Markman et al., 1993; McMullen, 1997; McMullen & Markman, 2000; Roese, 1994, 1997; Roese & Olson, 1995a, 1995b, 1995c; Taylor & Schneider, 1989).

## Counterfactuals in Gambling

In the context of gambling, the spontaneous tendency to generate UCTs following a loss may be used to alert gamblers to negative outcomes and assist in identifying corrective and/or preventative strategies (e.g., setting a budget, time limit). Learning from past mistakes may assist in avoiding future losses. On the other hand, generating DCTs after winning may enable gamblers to gain appreciation for their success in avoiding a worse outcome. This may encourage gamblers to savor their win and promote more risk-aversive behavior focused on protecting returns gained.

Markman et al. (1993) were among the first to demonstrate the preparative function of UCTs in a gambling study using a computer simulated game of blackjack. They found participants generated more UCTs when losing or when expecting to play again because UCTs can provide useful information on necessary changes or corrections believed to improve outcomes. However, while UCTs provide preparative benefits, some have found that UCTs can negatively impact outcome evaluations via a contrast effect resulting in increased negative affect by virtue of comparison with the actual outcome obtained (Schwarz & Bless, 1992; Sherif & Hovland, 1961). Consequently, UCTs may leave the individual prepared but feeling worse (Markman et al., 1993; Roese, 1994).

Based on this theorizing, we believe that the more usual pattern of generating UCTs after losses and DCTs after winning can support infrequent gamblers conservative and potentially more responsible approach to gambling. Specifically, infrequent gamblers encountering a loss benefit from UCTs, because these thoughts allow the gambler to learn from the past and thus prepare to make changes in their future playing behavior. For infrequent gamblers, this may include implementing preventative playing strategies (e.g., setting a budget, time limits) and corrective actions to avoid similar future failures. In addition, the negative affect evoked in response to generating UCTs could provide information that supports more cautious preparation efforts. Specifically, negative affect evoked by UCTs can provide information that the activity is problematic and potentially threatening (Bless et al., 1992; Schwarz, 1990, 2012). As such, we propose that the increased negative affect elicited by UCTs is consistent with infrequent gamblers more conservative gambling approach. This is because heightened attention to a situation’s threatening cues promotes decisions favoring risk-aversion and encourages more vigilant and cautious future behavior (Mayer & Hanson, 1995; Schulreich et al., 2016; Schwarz & Clore, 1983; Wright & Bower, 1992; Yuen & Lee, 2003).

Following a win, and in line with their affective function, we argue that DCTs resulting in positive affect can allow infrequent gamblers to appreciate successes which could have easily been worse (i.e., a loss). Accordingly, DCTs should function to enable infrequent gamblers to savor their win and reduce the desire to keep playing to protect gains. Consistent with this hypothesis, Wu et al. (2017) found that near losses elicited more DCTs (conceptualized as a positive outcome, e.g. “At least I won, I could have gone bankrupt!”) and less intent to gamble. In the near loss operationalized by Wu et al. (2017), the gambler saw the loss as the wheel slowed down, but eventually won. In this setting the counterfactual is the possible loss that could have eventuated, but thankfully didn’t. Further, more recently, Awo et al. (2023) found an association between wins and the generation of DCTs which reduced the intention to bet further in line with a posited ‘resource protective’ role.

While the usual pattern of generating UCTs after a loss, and DCTs after a win can support infrequent gamblers more conservative gambling approach, we propose that this pattern of counterfactual thinking may interfere with frequent gamblers’ more eager gambling approach because the affective costs of UCTs after a loss and the preparative costs of DCTs after a win, will make it harder for frequent gamblers to justify continued gambling. For example, spontaneously generating UCTs after a loss may be useful because they will help frequent gamblers to identify corrective strategies regarding how the outcome could be improved via the preparative benefits provided. However, increased negative affect associated with UCTs could magnify perceptions of threat and reduce optimism which could leave frequent gamblers feeling discouraged and make it harder to justify that it is valuable to continue playing. Further, the tendency to generate DCTs as an immediate response after a win may be beneficial because they enhance enjoyment for the win and can allow frequent gamblers to appreciate and feel good about their success navigating worse outcomes via the affective benefits they provided. However, while DCTs may lead to immediate feelings of satisfaction, they have been characterized as detrimental to motivation because the positive feelings evoked can lead to complacency after success and suggest that further action is not required (Markman et al., 2008; McCrea, 2008; McMullen & Markman, 2000). Therefore, lingering positive feelings after a gambling win could in turn cause complacency after success and will not provide the motivation to pursue more opportunities to win or help frequent gamblers identify how to further improve on their winnings. In the next section, we discuss how frequent gamblers may be motivated to generate dual counterfactuals to help support their beliefs that continued gambling to chase wins and losses is beneficial.

## Dual Counterfactuals May Help Justify Continued Playing

We argue that dual counterfactuals are generated to counteract the costs of more immediate counterfactual responses and provide frequent gamblers with the combination of preparative and affective benefits needed to justify risky decision making and potentially more irresponsible gambling behavior. Specifically, we propose that when frequent gamblers lose, similar to infrequent gamblers, UCTs will be useful because they can help identify corrective actions and preparative strategies to avoid similar mistakes and improve future outcomes. For frequent gamblers, who are eager to continue playing, the focus may be on identifying corrective strategies (e.g., information on differences in volatility—return to player percentages—between electronic gaming machines, multi-coin-multi-line strategies) that may improve the potential for increasing returns.

Frequent gamblers could even be motivated to use UCTs for preparative benefits in gambling situations where outcomes are determined primarily by luck and where learning may be difficult. This is because frequent gamblers are more vulnerable to illusions of control (Källmén et al., 2008; Ladouceur et al., 2003; Orgaz et al., 2013) and therefore, more likely to believe that they understand and can control causal factors to success identified via UCT generation (Petrocelli & Sherman, 2010). Further, we argue that in addition to generating UCTs of how the outcome could have been better to gain preparative benefits through changing their strategy of play, they will also consider how the outcome could have been worse. Frequent gamblers need to generate DCTs, because the positive feelings elicited can mitigate the affective costs of UCTs and help diminish the sense of perceived threat highlighted by increased negative affect evoked in response to UCTs. Supporting this perspective, positive affect, and feelings of optimism about the chances of winning have been identified as playing a critical role in problem gambling relapse (Hodgins & el-Guebaly, 2004; Holub et al., 2005). Therefore, through their affective benefits, DCTs will enable frequent gamblers to feel better about a loss and can provide a source of comfort and self-enhancement that can be used by frequent gamblers to maintain their rosy outlook and support their belief that they can win again if they keep playing.

**Fig. 1:**
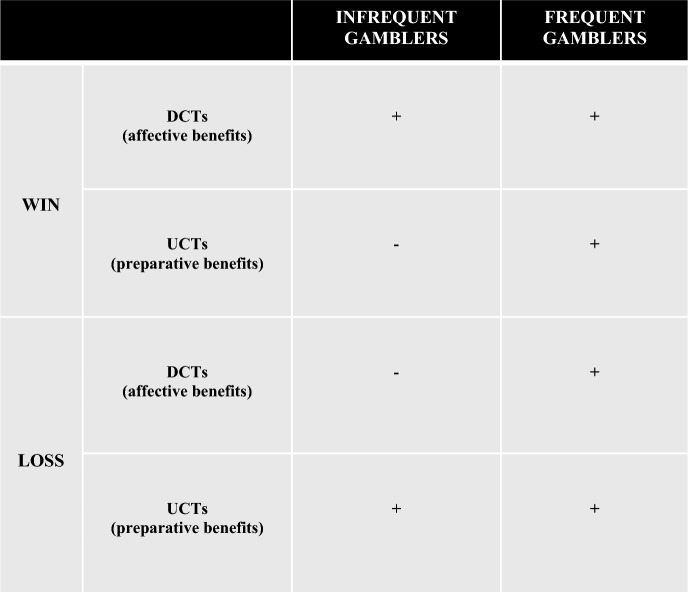
Conceptual summary

When frequent gamblers win, similar to infrequent gamblers, DCTs should be useful because they will allow frequent gamblers to appreciate their ability to avoid an alternative outcome which could have easily been worse and can enhance immediate enjoyment for their success via the affective benefits provided. However, to avoid becoming complacent and support their eagerness to keep playing, we expect that they will also consider how the outcome could have been better. Frequent gamblers need to generate UCTs to mitigate the preparative deficits of DCTs and to remind themselves that there are still opportunities to do better and to identify how they may be able to improve in order to keep winning. Refer to Conceptual Summary in Fig. 1.

## Hypotheses

Given that counterfactual thoughts are often evaluative in nature (Epstude & Roese, 2008), an intriguing and unexplored question is whether counterfactuals thoughts are subject to similar biases among individuals experiencing problem gambling? If so, how might counterfactual thoughts in this subgroup shift from the typical and often spontaneous pattern of generating UCTs after losing, and DCTs after winning, to an alternative pattern justifying more risky decision-making and desire to continue to chase losses and wins? We advance the hypothesis that as gambling frequency increases, gamblers will generate ‘dual counterfactuals’. We define dual counterfactuals as generating a pattern of both UCTs *and* DCTs in response to an obtained outcome.

We propose that this atypical pattern of dual counterfactuals is generated by frequent gamblers to counteract the costs associated with more usual counterfactual responses (i.e., UCTs after losing, and DCT’s after winning). In this context, the addition of DCTs after losing, and UCTs after winning can provide the necessary affective and preparative benefits to justify the frequent gamblers’ desire to continue playing.

We focus on frequent (vs. infrequent gamblers) because gambling frequency has been identified as a possible risk factor for problem gambling (Currie et al., 2006, 2008). Consistent with our theorizing, we hypothesize that infrequent gamblers will show a significant difference in the direction of their counterfactual thoughts for a loss compared to a win (more UCTs for loss, more DCTs for win), and that they should feel more upset with a loss relative to a win. In contrast, we hypothesize that unlike infrequent gamblers, frequent gamblers will not show a significant difference in the direction of their counterfactual thoughts for a loss compared to a win (both UCTs *and* DCTs will be equally present regardless of outcome). We also propose that the dual counterfactual response generated should impact frequent gamblers emotional response to the outcome obtained such that the generation of UCTs (which elicit negative affect) and the generation of DCTs (which elicit positive affect), should result in no significant differences in how upset (and/or how happy) they feel about a loss relative to a win.

Additionally, regarding expected differences *across* gambler type, following a loss, we expect that both frequent and infrequent gamblers will equally generate UCTs to gain preparative benefits, but that frequent gamblers will generate more DCTs than infrequent gamblers to counteract UCTs affective costs. Consequently, we hypothesize that frequent gamblers will feel relatively less upset (and/or happier) following a loss compared to infrequent gamblers. Following a win, we expect both frequent and infrequent gamblers will gain immediate affective benefits following the generation of DCTs, but that frequent gamblers will generate more UCTs than infrequent gamblers to motivate and justify continued play to pursue better opportunities. Consequently, the shift to a preparative mode for frequent gamblers will result in them feeling relatively less happy following a win compared to infrequent gamblers.

In the following section we outline our study design and method. We test our hypotheses regarding the dual counterfactuals generated by frequent gamblers in a simulated poker machine experiment amongst a sample of gamblers in a real club setting. We also explore gamblers’ affective reactions in order to gain insight into the means through which dual counterfactuals may exert their influence.

## Method

### Participants and Design

One hundred and forty-two gamblers participated in the study. Participants were recruited via posters in Returned Service League Clubs where activities, including the type of poker machine which the Maid Marion game is designed to mimic, are typically located. The design included one manipulated independent and one measured factor. The patterns of wins and losses in the games were designed to manipulate the outcome of the experience (win, loss). Questions were asked at the end of the study to measure whether the participant was a frequent gambler. Four participants did not indicate their gambling frequency, and therefore could not be included in the analyses. Therefore, a usable sample of 138 field gamblers remained.

### Procedure

**Fig. 2:**
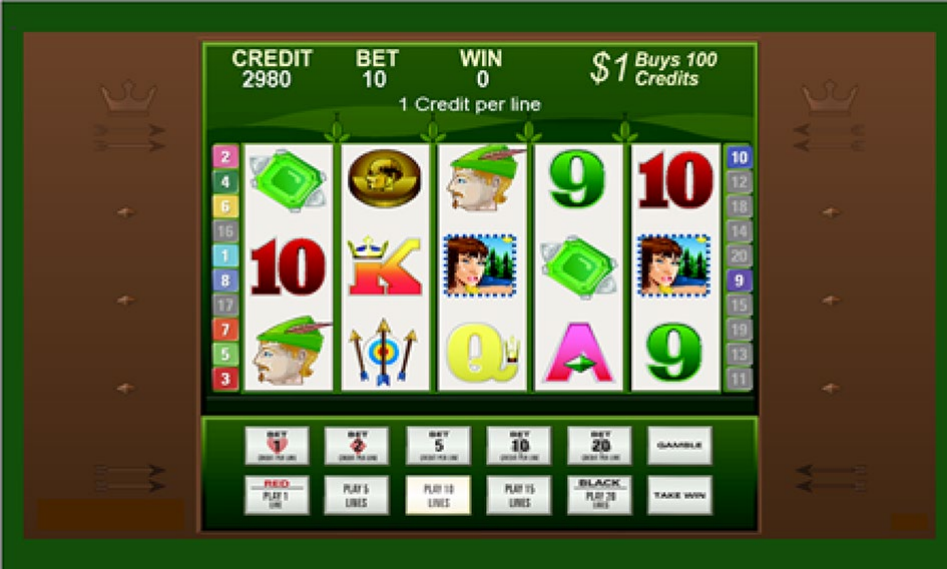
The maid Marion game screen

The study was run at the clubs in a room located near the gaming floor. The cover story used indicated that the study investigated peoples’ perceptions and opinions of a new gambling game. After signing an informed consent, participants were lent, not given, credits to begin the game. Participants were lent credits to ensure they played the game instead of using the credits for another activity. Participants played 300 games on a laptop computer displaying a simulated poker machine game called Maid Marion that was designed to look and sound like a gaming machine. The game comprised of five wheels with three rows. Each spin resulted in a matrix of 15 symbols (refer to Fig. 2). The symbols unique to this game were Maid Marion, Robin Hood, a shield, a bow and bull’s eye, a ring, a gold coin, and a castle. Other symbols included the standard card faces of an ace, king, queen, jack, ten, and nine. Although the participants were led to believe that the outcome of each individual game was random, the exact pattern of wins and losses was pre-determined.

Participants played 300 games in total. At the end of 300 games, participants were awarded the financial equivalent of 1¢ for each credit above the original number of credits. The patterns of outcomes for the gambling sessions were designed such that half the participants won, and half lost. Winners won approximately 2000 credits ($20). To ensure the cover story was feasible, small variations in the patterns were included such that not all winners received exactly the same amount. Losers lost approximately 2000 points. In addition to the credits won during the game, at the end of the study, all participants were debriefed and awarded $20 of club membership points to compensate them for their time in completing the gambling study session.

### Independent Measure

#### Gambling Frequency

A measure of gambling frequency was calculated using a composite of two questions. Firstly, participants were asked how often they played gaming machines in the past 12 months on a nine-point scale (*M* = 4.57, *Mdn* = 4.00, *SD* = 2.69) anchored by less than twice a year (1), once every 4 to 6 months (2), once every 2 to 3 months (3), once a month (4), 2–3 times a month (5), once a week (6), twice a week (7), three times a week (8) to four times a week or more (9). Secondly, participants were asked how long they played gaming machines on a typical visit on an eight-point scale (*M* = 3.57, *Mdn* = 4.00, *SD* = 1.75) anchored by less than 10 min (1), 10 min to 30 min (2), 31 min to 1 h (3), 1 h to 2 h (4), 2 h to 3 h (5), 3 h to 5 h (6), 5 h to 8 h (7), to more than 8 h (8). See “Appendix”. The two measures were standardized and summated to form a single measure of gambling frequency* r* = 0.33, *p* < 0.001.

### Dependent Measures

#### Counterfactual Thoughts

Adapted from previous research (Rim & Summerville, 2014), after the game ended upward counterfactuals were measured by asking participants to indicate on a 100 mm continuous scale the extent to which the outcome could have been better (*M* = 68.59*, SD* = 31.07) with one end of the scale anchored “not at all better” and the other end of the scale anchored “much better” (see “Appendix”). To measure downward counterfactuals, participants were asked to indicate on a 100 mm continuous scale the extent to which the outcome could have been worse (*M* = 67.81*, SD* = 32.29) with one end of the scale anchored “not at all worse” and the other end of the scale anchored “much worse”.

#### Affective Reactions

Participants were also asked to indicate how they felt about the outcome by reporting the extent to which they felt disappointed, anxious, and pleased using 100 mm continuous scales (see “Appendix”). The entire procedure took approximately 40 min.

## Results

### Counterfactual Thoughts

Within our experimental design all participants were asked to report both UCTs and DCTs to allow the use of a repeated measures approach in the analysis. Based on established practice of using median splits in the gambling literature (Gainsbury et al., 2013; Hearn et al., 2021) and following statistical criteria satisfying the appropriate use of median splits more broadly (Iacobucci et al., 2015),^1^ we first categorized recruited gamblers into infrequent and frequent gambling groups based on the median of the two standardized and summated frequency questions. Those scoring above or at the median of the summated frequency measure (*Mdn* = 0.025; *SD* = 0.82) were classified as ‘frequent’ gamblers, while those who scored below the median were classified as ‘infrequent’ gamblers. Next, we conducted a mixed model ANOVA analyses with outcome (win, loss) and gambler type (frequent, infrequent) as between-subjects variables and counterfactual direction (upward, downward) as the repeated measure. The results revealed a significant outcome × counterfactual direction interaction *F* (1, 134) = 24.40, *p* < 0.001. Importantly, this was qualified by a significant outcome × gambler type × counterfactual direction interaction *F* (1, 134) = 8.16, *p* = 0.005.

To explore the nature of the 3-way interaction, we first examined relative differences in the counterfactual direction of thoughts within gamblers by examining the outcome by counterfactual direction interaction separately for each gambler type. Infrequent gamblers generated more upward (*M* = 79.59, *SE* = 5.00) than downward counterfactual thoughts following a loss (*M* = 52.68, *SE* = 5.37), *F* (1, 134) = 17.04, *p* < 0.001, and more downward (*M* = 73.26, *SE* = 5.29) than upward counterfactual thoughts following a win (*M* = 49.49, *SE* = 4.92), *F* (1, 134) = 13.69, *p* < 0.001. Alternatively, for frequent gamblers there were no significant differences in the proportion of upward relative to downward counterfactuals for each type of outcome. Specifically, frequent gamblers generated an equal extent of upward versus downward counterfactual thoughts following a loss (*M*_*upward*_ = 75.60, *SE* = 5.32 vs. *M*_*downward*_ = 67.60, *SE* = 5.71), *F* (1, 134) = 1.33, *p* = 0.251 and a win (*M*_*upward*_ = 70.74, *SE* = 4.66 vs. *M*_*downward*_ = 76.28, *SE* = 5.01), *F* (1, 134) = 0.828, *p* = 0.365.

**Fig. 3:**
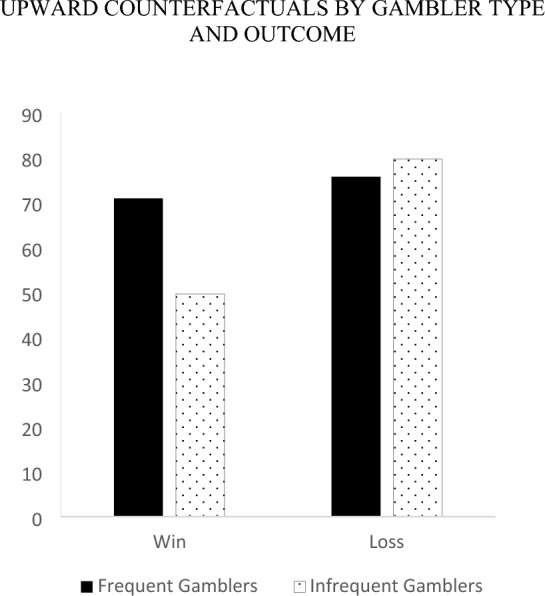
Upward counterfactuals by gambler type and outcome

**Fig. 4:**
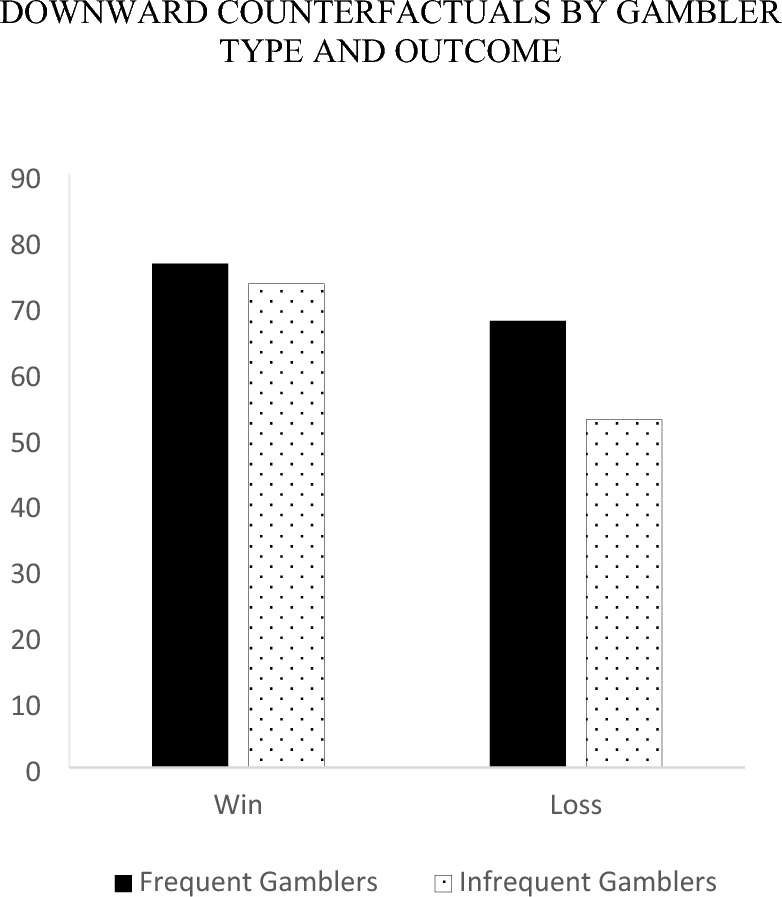
Downward counterfactuals by gambler type and outcome

Next, we compared differences in the counterfactual direction of thoughts across gamblers by examining the gambler type by counterfactual direction interaction separately for each outcome. Following a loss, frequent and infrequent gamblers generated an equal extent of upward counterfactuals (*M*_*frequent*_ = 75.60, *SE* = 5.32 vs. *M*_*infrequent*_ = 79.59, *SE* = 5.00), *F* (1, 134) = 0.30, *p* = 0.586. Additionally, after losing, frequent gamblers generated marginally more downward counterfactuals (*M* = 67.60, *SE* = 5.71) than infrequent gamblers (*M* = 52.68, *SE* = 5.37), *F* (1, 134) = 3.63, *p* = 0.06. Following a win, frequent (*M* = 76.28, *SE* = 5.01) and infrequent gamblers (*M* = 73.26, *SE* = 5.29) generated an equal extent of downward counterfactuals *F* (1, 134) = 0.17, *p* = 0.68. Furthermore, after winning, frequent gamblers generated more upward counterfactuals (*M* = 70.74, *SE* = 4.67) than infrequent gamblers (*M* = 49.49, *SE* = 4.92), *F* (1, 134) = 9.82, *p* = 0.002. See Figs. 3 and 4.

### Affective Reactions

**Fig. 5:**
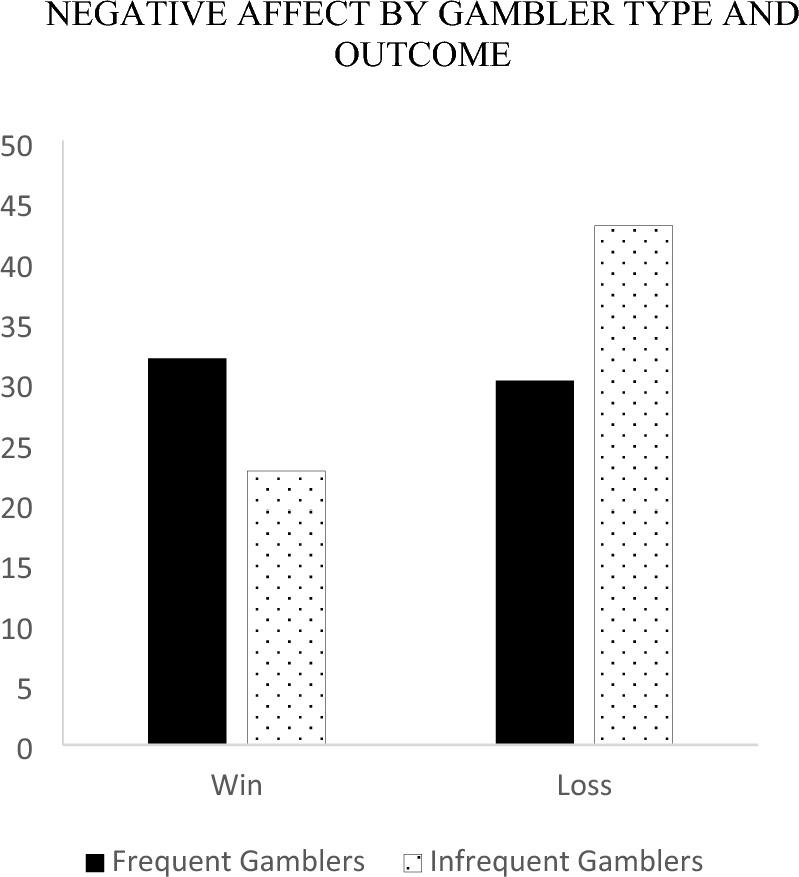
Negative affect by gambler type and outcome

To examine how gamblers counterfactual thoughts mapped onto affect, a 2 outcome (win, loss) by 2 gambler type (frequent, infrequent) between-subjects ANOVA was run on each of the affective reaction variables. Firstly, we examined the negative affect ratings. The measure of disappointment and anxiety were correlated (*r* = 0.291;* p* < 0.001) and summated to form a single measure of negative affect (*M* = 31.95, *SD* = 24.57). The results revealed a significant main effect *F* (1, 133) = 5.15, *p* = 0.025 for outcome indicating that a loss (*M*_*loss*_ = 36.52, *SE* = 2.97) resulted in more negative feelings than a win (*M*_*win*_ = 27.28, *SE* = 2.78).^2^ This result was further qualified by a significant outcome × gambler type interaction *F* (1, 133) = 7.40, *p* = 0.007. Follow-up simple effects revealed that following a loss, infrequent gamblers (*M* = 42.94, *SE* = 4.07) were significantly more upset than frequent gamblers (*M* = 30.10, *SE* = 4.33), *F* (1, 133) = 4.67, *p* = 0.033. Following a win, frequent gamblers (*M* = 31.94, *SE* = 3.80) were marginally more upset than infrequent gamblers (*M* = 22.62, *SE* = 4.07), *F* (1, 133) = 2.80, *p* = 0.097. Furthermore, simple effects analyses revealed that for infrequent gamblers, a loss generated significantly more negative feelings (*M* = 42.94, *SE* = 4.07) than a win (*M* = 22.62, *SE* = 4.0), *F* (1, 133) = 12.47, *p* < 0.001. In contrast for frequent gamblers, there were no significant differences in negative affect for a loss (*M* = 30.10, *SE* = 4.33) relative to a win (*M* = 31.94, *SE* = 3.80), *F* (1, 133) = 0.101, *p* = 0.751 (see Fig. 5).

**Fig. 6:**
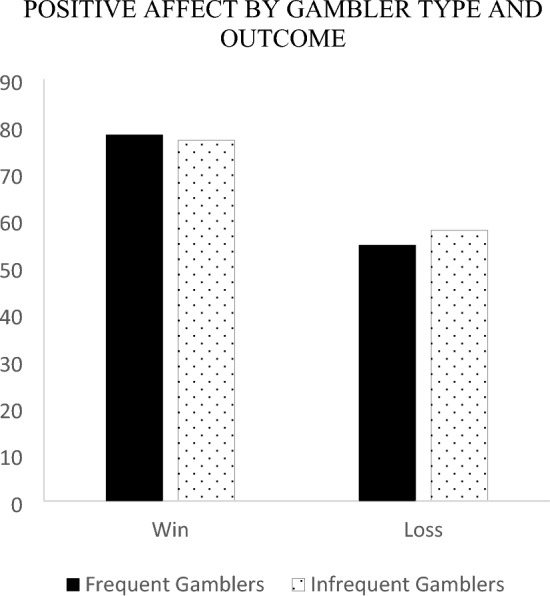
Positive affect by gambler type and outcome

Next, we examined the positive affect rating of pleasure (*M* = 67.62, *SD* = 26.12) to gauge the extent of positive affect. A main effect of outcome emerged such that participants reported feeling more pleased after a win (*M* = 77.54, *SE* = 2.83) compared to a loss (*M* = 56.17, *SE* = 3.02), *F* (1, 133) = 26.73, *p* < 0.001. There were no other significant main effects or interaction *F*’s < 1. See Fig. 6.

## Discussion

As hypothesized, we found that infrequent gamblers generated UCTs after losing and DCTs after winning, while frequent gamblers generated both UCTs *and* DCTs following a loss and win. Furthermore, frequent gamblers felt less upset than infrequent gamblers after a loss, and more upset than infrequent gamblers after a win. Additionally, for infrequent gamblers a win generated fewer negative feelings than a loss. However, for frequent gamblers a win generated similar levels of negative affect to a loss. The pattern of affect supports the idea that after a loss, the affective costs of UCTs may be a hindrance to frequent gamblers. Accordingly, frequent gamblers may use DCTs to maintain a positive outlook after a loss and to help soften the impact of helpful, but negatively charged UCTs. For a win, frequent gamblers may generate UCTs to counter the preparative costs of DCTs and to avoid the complacency which may creep in after winning to ensure they stayed focused on improving and pursuing bigger and better opportunities.

### Contributions

Our research contributes to the growing but limited body of gambling research exploring how gamblers engage in cognitive processes, such as counterfactual thinking to evaluate their gaming outcomes. Further our findings build on the limited work examining motivated biases amongst gamblers. Previous research has found some evidence for the generation of UCTs following losses, and DCTs following wins amongst non-problem population of gamblers^3^ (Awo et al., 2023; Wu et al., 2017). These findings mirror the pattern of counterfactual thinking we found for infrequent gamblers in our sample. We extended this work by focusing on the unexplored counterfactual processes of frequent gamblers and identified gambling frequency as an important moderator for the tendency to gain motivational utility from a dual pattern of dual counterfactuals. Through this focus, our findings provide a novel demonstration of how frequent gamblers may engage in motivated reasoning in the form of generating dual counterfactuals to justify risky, and potentially irresponsible gambling behavior.

Our findings also contribute to psychological theorizing on the processes through which counterfactual thinking can influence subsequent motivation and preparatory behavior. Epstude and Roese (2008) distinguished between a content-neutral pathway and a content-specific pathway as mechanisms underlying the motivational function of counterfactual thinking. In the case of the content-specific pathway, behavioral intentions are facilitated directly from the content of the counterfactual itself activating functional links to goal-directed behavior in memory (Smallman & Roese, 2009). A counterfactual identifies possible causal inferences, which translate into specific strategies a person intends to perform, in order to bring about improvement in the strategy of play and, possibly, the outcome.

In the content-neutral pathway, counterfactuals may influence behavior independently of the semantic content provided by the counterfactual. The counterfactual may ignite motivations including affect, self-inferences, or mind-sets that themselves fuel behavior. Importantly, the theory holds that the components of the content-neutral pathway may operate independently to or interactively with the information contained within the counterfactual. The results of the present set of studies extend this work by showing that frequent gamblers may use an interactive pathway via the dual counterfactuals they generate. Specifically, UCTs may offer valuable information specifying means to improve in the future via the rich content they provide. In addition, DCTs through their affective benefits offer a valuable source of positive affect that enables self-enhancement.

### Limitations

There are several methodological limitations that should be considered when interpreting the findings and designing future research. First, we theorized and found that both infrequent and frequent gamblers appear to benefit from generating UCTs due to the preparative information provided. We also highlighted the role of gambling frequency as a potential moderator of the risk factor associated with UCTs (Awo et al., 2023), by proposing that UCTs may differentially impact the propensity to take risk and engage in potentially maladaptive gambling behavior depending on the type of UCT content generated. However, we did not measure the specific content of their UCTs, and the types of strategies generated. Future research would benefit from examining which corrective strategies infrequent and frequent gamblers share, and which strategies are unique to each gambling population. For example, in terms of commonalities, it is possible that both gambler types benefit from generating UCTs focused on gaining better knowledge of the game mechanics and rules to avoid future errors. In terms of differences, infrequent gamblers may be more focused on identifying preventative strategies such as setting budgets and time limits to try to minimize future losses, while frequent gamblers may be more focused on identifying eager strategies such as exploring games features and bonuses to try to boost their wins.

Second, we focused and measured low-arousal negative emotions such as anxiety and disappointment, which may have provided a stronger signal of threat (MacLeod & Matthews, 1988) and thereby may have heightened the affective costs of UCTs for frequent gamblers after a loss. However, it is possible that in some gambling contexts and following certain outcomes, high arousal negative emotions such as anger may be more prominent. In this case, signals of threat would be lowered and elevated feelings of frustration and anger may directly encourage more risk seeking behavior (Lerner & Keltner, 2001). Future research would benefit from examining appraisal-tendency based differences in the specific type of affect generated as a function of counterfactual direction.

Thirdly, participants were requested to play with credits provided to them, as opposed to risking their own money, a research design that is limited by ethical considerations. However, there do not appear to be any a priori reasons to suggest that differences in findings would emerge between the credit provided and use of own money design.

Despite these limitations, the current study provides new novel insights into the counterfactual thinking and affect patterns experienced by gamblers as a function of their playing frequency and sets the stage for continued research in this nascent area.

### Clinical and Service Implications

Building on work demonstrating the benefits of using cognitive restructuring techniques to help correct potentially erroneous, dysfunctional or inadequate gambler-related thoughts (e.g. Blaszczynski et al., 2001; Chretien et al., 2017; Ladouceur et al., 2001, 2003), our findings suggest that there may be clinical value in challenging frequent gamblers’ counterfactual thinking processes through educational interventions, public health initiatives and gambling advertisements/announcements to interrupt potentially disruptive “what-if” thoughts and help encourage more responsible gambling behavior. For example, problem gambling support services may not only benefit from incorporating gambling educational tools to enable gamblers to gain a better gauge of resources (e.g. time, money) spent on gambling activities. These could also include self-assessment quizzes and complementary cognitive reframing strategies to help frequent gamblers replace UCTs after winning with more DCTs focused on appreciating wins and protecting returns gained. This can be achieved by highlighting the potential for worse outcomes. It could further assist frequent gamblers to replace DCTs after losing with more UCTs focused on identifying preventative strategies (e.g., setting a budget, time limit) aimed at learning from past mistakes to avoid future losses. Additionally, public health service and gambling advertisements could incorporate evidence-based warning messages with mottos and taglines focused on questioning and dissipating counterfactual thoughts based on risky, and potentially damaging “what-if” possibilities.

## Conclusions

The current study presents a novel examination of the counterfactual thinking patterns of frequent gamblers. Unlike infrequent gamblers who exhibit a more typical pattern of UCTs following losses, and DCTs following wins, the current research finds that frequent gamblers generate a pattern of ‘dual counterfactuals’ – comprising of both UCTs and DCTs – following both wins and losses. The results of this study identify dual counterfactuals as a distinct form of motivated reasoning that frequent gamblers may use to justify the desire to continue gambling for better *and* for worse. The results of the project can be used to inform cognitive restructuring techniques and clinical, educational and public health interventions which challenge potentially disruptive counterfactual “what-if” thoughts to help minimize gambling related harm.

## Data Availability

The data that support the findings of this study are available from the corresponding author upon reasonable request.

## Appendix: Survey Measures

### Counterfactual Thinking

The outcome could have been (please place an X on the scale to indicate your response):

The outcome could have been (please place an X on the scale to indicate your response):

### Affective Reactions

How do you feel about the outcome of the gambling game?

(Please place an X on each scale to indicate your feeling).

### Gambling Frequency Measures

How often have you played gaming machines in the past 12 months?

How long do you generally play the gaming machines on a typical visit?

## Footnotes

1 The correlation between the experimental factor (outcome) and the median split variable (gambling frequency) was zero; H0: ρ = 0; r = .058, *p* = .498 (*NS*).

2 One participant completed the counterfactual ratings but did not complete the affective ratings, resulting in one less degree of freedom.

3 In Awo et al. (2023), participants were further screened for problem gambling using the SOGS-RA. Participants reporting mild to moderate problem gambling were excluded from the study.
